# Amyloid-β Inhibits No-cGMP Signaling in a CD36- and CD47-Dependent Manner

**DOI:** 10.1371/journal.pone.0015686

**Published:** 2010-12-22

**Authors:** Thomas W. Miller, Jeff S. Isenberg, Hubert B. Shih, Yichen Wang, David D. Roberts

**Affiliations:** 1 Laboratory of Pathology, Center for Cancer Research, National Cancer Institute, National Institutes of Health, Bethesda, Maryland, United States of America; 2 Department of Medicine, University of Pittsburgh School of Medicine, Pittsburgh, Pennsylvania, United States of America; 3 Howard Hughes Medical Institute–National Institutes of Health Research Scholars Program, Bethesda, Maryland, United States of America; Federal University of Rio de Janeiro, Brazil

## Abstract

Amyloid-β interacts with two cell surface receptors, CD36 and CD47, through which the matricellular protein thrombospondin-1 inhibits soluble guanylate cyclase activation. Here we examine whether amyloid-β shares this inhibitory activity. Amyloid-β inhibited both drug and nitric oxide-mediated activation of soluble guanylate cyclase in several cell types. Known cGMP-dependent functional responses to nitric oxide in platelets and vascular smooth muscle cells were correspondingly inhibited by amyloid-β. Functional interaction of amyloid-β with the scavenger receptor CD36 was indicated by inhibition of free fatty acid uptake via this receptor. Both soluble oligomer and fibrillar forms of amyloid-β were active. In contrast, amyloid-β did not compete with the known ligand SIRPα for binding to CD47. However, both receptors were necessary for amyloid-β to inhibit cGMP accumulation. These data suggest that amyloid-β interaction with CD36 induces a CD47-dependent signal that inhibits soluble guanylate cyclase activation. Combined with the pleiotropic effects of inhibiting free fatty acid transport via CD36, these data provides a molecular mechanism through which amyloid-β can contribute to the nitric oxide signaling deficiencies associated with Alzheimer's disease.

## Introduction

The pathogenesis of Alzheimer's disease is closely associated with the accumulation of amyloid-β (Aβ) peptides, which eventually form neuronal deposits known as senile plaques on the outside surface of the neurons [1] and lead to neuron death. Aβ is a peptide of 37–43 amino acids in length that originates by proteolytic cleavage from the amyloid precursor protein, which is a neuronal transmembrane protein that contributes to innate antimicrobial immunity and has unknown function in the CNS [2]. Binding of Aβ to the plasma membrane is thought to be a critical step in development of Alzheimer's disease [3], and the formation of Aβ plaques is a primary trigger of neuron degeneration [4]. However, our molecular understanding of how Aβ contributes to the pathogenesis of Alzheimer's disease remains incomplete [5], [6], [7].

Nitric oxide (NO) is a cell signaling molecule that plays an important role in regulating vascular, immune, and neurological processes. For example, both hippocampal and cortical long-term potentiation, a physiological correlate of synaptic plasticity thought to underlie learning and memory, involve NO signaling cascades [8], [9]. NO can originate from exogenous sources and diffuse across the cell membrane, or it can be synthesized from L-arginine within the cell by nitric oxide synthases (NOS). NO activates soluble guanylate cyclase (sGC) to produce cGMP [10], which activates cGMP-dependent kinase (cGK), a major cellular receptor of cGMP [11]. cGK then catalyzes the phosphorylation of its substrates, which initiate various cellular responses such as smooth muscle relaxation, delayed platelet aggregation, intestinal secretion, and long term potentiation [12], [13], [14], [15].

NO in the brain can be produced either by inducible NOS (iNOS/NOS2) in microglia and astrocytes, or by constitutive NOS in neurons and endothelial cells (nNOS/NOS1 and eNOS/NOS3). A large body of evidence suggests that the NO produced by neuronal and endothelial constitutive NOS is responsible for neuroprotection during Aβ-induced cell death, while NO production in the case of iNOS activation plays a neurotoxic role due to the inflammatory response caused by the over generation of other reactive nitrogen species from NO (see review [16]). A decrease in neuronal NOS and an increase in hippocampal iNOS have been demonstrated in aged rats [17], thus suggesting the dual roles of NO. In mice, the higher level of constitutive NO produced by iNOS protects beta-amyloid transgenic mice from developing most typical human symptoms of Alzheimer's disease [18]. When crossed into an iNOS-null background these mice displayed extensive tau pathology associated with regions of dense microvascular amyloid deposition.

The protective role of NO in Alzheimer's disease pathogenesis has been linked to NO/sGC/cGMP/cGK signaling cascades. Treatment with NO donors and cGMP analogues suppresses cell death [19], and increasing intracellular cGMP levels prevents inflammatory responses in brain cells [20]. Moreover, the use of the NO donors, sGC stimulators, and cGMP-analogs reverses learning and memory impairment through cGK activation, in part by reestablishing the enhancement of the transcription factor cAMP-responsive element-binding protein (CREB), which is phosphorylated during long term potentiation [21].

However, an accumulation of Aβ inhibits the NO signaling pathway and therefore may suppress the protective effects of endogenous NO in the brain. Chronic administration of fibrillar Aβ decreases the expression of sGC in cultured rat astrocytes, desensitizing them to treatment with sodium nitroprusside [22]. Acute Aβ administration blocks NO-induced vasoactivity in rats [23], [24] and inhibits NO-stimulated phosphorylation of CREB [21]. The molecular mechanisms behind the down regulation of NO signaling by acute Aβ exposure remain a mystery, and are the focus of this paper. Understanding these mechanisms can potentially provide the basis for a novel therapeutic application of drugs aimed at limiting the adverse effects of Aβ.

The well-studied inhibitor of NO-cGMP signaling thrombospondin-1 (TSP1) shares several features with Aβ that suggested a common mechanism to inhibit NO signaling. Among the known cell surface TSP1 receptors, three have been proposed to also interact with Aβ: CD36, CD47, and α_6_β_1_ integrin [25], [26]. Although direct binding of Aβ to each of these receptors has not been established, the ability of Aβ to stimulate interleukin-1β release and reactive oxygen species production in microglial cells was inhibited by antibodies to each receptor [26]. Because CD36 and CD47 are each known to associate with some β_1_ integrins, the authors proposed that Aβ binds to a complex of these 3 receptors. Microglial phagocytosis of Aβ fibrils was subsequently shown to be inhibited by antagonists of the same receptor [27]. A GST-CD36 fusion protein and a CD47-binding peptide inhibited Aβ-stimulated Vav1 phosphorylation in THP-1 monocytes [28]. Furthermore, the ability of Aβ to stimulate histamine release by mast cells was inhibited by antibodies against CD47 and β_1_ integrin subunit and by a modified TSP1 peptide that binds to CD47 [29]. Contrary to these receptors recognizing Aβ as a complex, phagocytosis of Aβ was enhanced by conditions that increased CD36 but decreased CD47 recruitment to lipid raft domains of microglial cells [30].

TSP1 inhibits NO signaling in vascular cells by binding to CD47 to decrease sGC activity and cGMP levels [25], [31], [32]. TSP1 binding to CD36 can also inhibit this pathway, but only in cells that also express CD47 [25]. To test whether Aβ suppresses sGC activity and thus inhibits NO signal transduction by the same mechanism as TSP1, we used intracellular cGMP production as an indicator of sGC activity after stimulation by NO. We demonstrate that inhibition of the NO-cGMP signaling pathway by Aβ requires CD47 and CD36 but may not involve a direct interaction of Aβ with CD47. Rather, CD47 signaling may be perturbed downstream of Aβ interacting with CD36.

## Results

### Aβ interacts directly with CD36 but not with CD47

**Figure 1:**
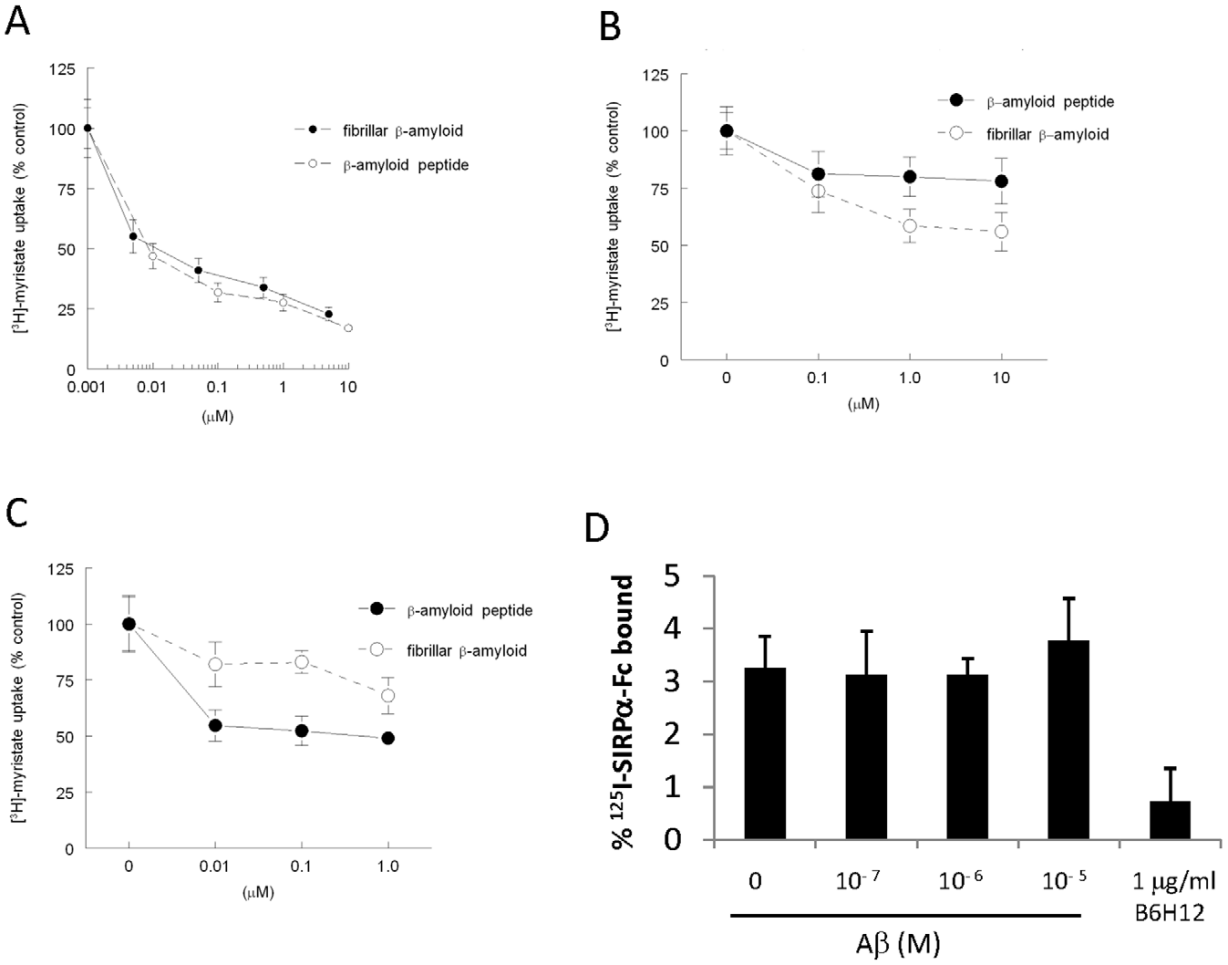
Fibrillar and soluble Aβ inhibit cellular myristate uptake but not SIRPα binding to CD47. [^3^H]-Myristic acid uptake after 5 min into human aortic VSMC (**A**), HUVEC (**B**), and microglial cells (**C**) was determined in the presence of the indicated concentrations of Aβ (soluble or fibrillar) after lysis by liquid scintillation counting. **D** ^125^I-SIRPα-Fc binding to Jurkat T-cells was measured in the presence of soluble Aβ (0.1-10 µM) or a CD47-specific function-blocking antibody (B6H12) for 1 hour at 25°C.

Aβ(1–42) has 3 predominate conformations, monomeric peptide, soluble oligomers, and fibrillar, that depend on the amount of time it is left in solution to aggregate. Controversy still exists as to which of these mediate Aβ pathology, and both the soluble and fibrillar forms are inhibitors of vascular responses [24], [33], [34], [35]. Considering this ambiguity, we compared the ability of fibrillar and soluble peptide forms to interact with CD36 based on their ability to inhibit the fatty acid translocase activity of CD36 [36], [37]. Both forms inhibited [^3^H]-myristic acid uptake by both glial and vascular cells in a dose-dependent manner (Fig. 1A–C). While their responses were equipotent in human aortic VSMC and HUVEC (Fig. 1A, B), the soluble peptide was more potent in microglial cells (Fig. 1C). Based on these results, we used the soluble peptide for the remainder of the studies.

Previous antibody and peptide inhibition studies implicated CD47 in Aβ signaling but did not determine whether Aβ binds directly to CD47 [26], [27], [28], [29]. The prototypical ligand for CD47 is the extracellular domain of its counter-receptor SIRPα [38]. We have previously shown that binding of the CD47 ligand TSP1 can be measured by displacement of labeled SIRPα-Fc fusion protein [39]. We employed the same assay to determine whether Aβ binds directly to CD47 but observed no significant inhibition of SIRPα-Fc binding at Aβ concentrations up to 10 µM (Fig. 1D). Therefore, the role of CD47 in mediating the inhibitory activity of Aβ may be indirect, as previously shown for peptide ligands of CD36 that also do not bind to CD47 [39].

Although we cannot exclude the possibility that Aβ indirectly inhibits the translocase activity of CD36, these data support a direct interaction between Aβ and the scavenger receptor CD36 but not with CD47.

### Soluble Aβ inhibits NO and BAY 41-2272 stimulated sGC activity

**Figure 2:**
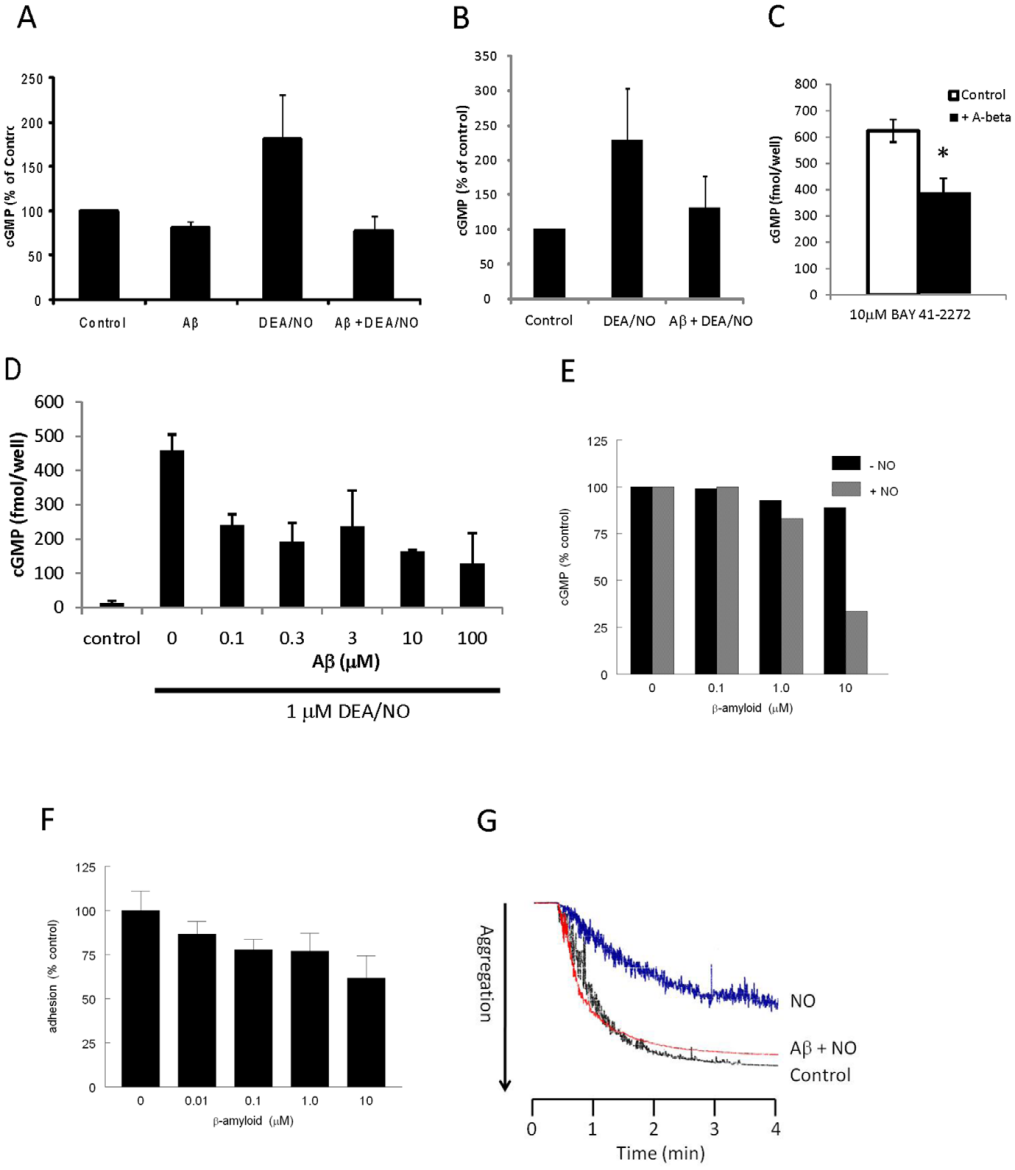
Aβ inhibits NO-induced cGMP synthesis and function. **A** BAEC were pretreated with 10 µM Aβ followed by 10 µM DEA/NO. **B** HUVEC were pretreated with 10 µM Aβ followed by 90 µM IBMX and 10 µM DEA/NO. **C** Jurkat cells were pretreated with 10 µM Aβ followed by an NO-independent sGC activator (BAY 41-2272). **D** Jurkat cells were pretreated with Aβ (0.1–100 µM) followed by 1 µM DEA/NO. **E** Porcine VSMC were pretreated with Aβ (0.1–10 µM) followed by 10 µM DEA/NO. Following treatment, cells were lysed and assayed for cGMP production. n = 3, * denotes P<0.05. **F** Porcine VSMC adhesion to collagen coated wells was assessed in the presence of Aβ (0.01–10 µM) for 1 hour. **G** Thrombin(0.2 U)-induced aggregation of washed human platelets was assessed in the absence (control) or presence of DEA/NO (0.01 µM) and with a 5 min pretreatment of Aβ (10 µM) followed by DEA/NO.

TSP1 engagement of CD47 or CD36 inhibits activation of sGC in several cell types [25], [32], [40], [41]. To evaluate the effect of Aβ on sGC activation, we tested whether Aβ directly blocks NO-stimulated cGMP production. Treatment of BAEC with 10 µM of Aβ led to a slight decrease in the basal levels of cGMP (Fig. 2A). As expected, addition of the fast-releasing NO donor DEA/NO (10 µM) caused a significant increase of cGMP production. Cells that were treated with Aβ followed by DEA/NO exhibited lower levels of cGMP than those given only the NO donor, suggesting that Aβ signaling directly blocks sGC activation. The cGMP data represented in Figure 1 were obtained without inclusion of a phosphodiesterase inhibitor such as 3-isobutyl-1-methylxanthine or sildenifil. Thus, the effect of Aβ on cGMP flux could result either from inactivation of sGC or stimulation of phosphodiesterase activity. However, Aβ also inhibited cGMP accumulation in the presence of 3-isobutyl-1-methylxanthine (IBMX, Fig 2B) establishing that Aβ signaling regulates sGC activation.

We recently reported that TSP1 also inhibits drug-induced activation of sGC [42]. Treatment of Jurkat cells with 10 µM of the synthetic sGC activator BAY 41–2272 led to an increase in cGMP production similar to that observed after treatment with DEA/NO (Fig. 2C). The increase intracellular cGMP induced by the BAY compound was inhibited by the addition of 10 µM of Aβ peptide. Therefore, Aβ suppresses both NO-induced and synthetic sGC stimulator-mediated sGC activation, reducing cGMP production and inhibiting NO signaling.

The inhibitory effect of Aβ on sGC activation extends to both Jurkat human T lymphoma cells (Fig. 2D) and porcine VSMC (Fig. 2E). A dose response in porcine VSMC revealed an IC_50_ value of about 5 µM Aβ, while the Jurkat T cells were more sensitive, having an IC_50_ of less than 100 nM. A dose of 10 µM Aβ was used in subsequent experiments as it caused a greater than 50% inhibition in all three cell types.

TSP1 inhibits NO/cGMP-stimulated VSMC adhesion on collagen [40], and we used this adhesion as a functional assay of the effect of Aβ on cGMP production. Paralleling its inhibition of cGMP production in primary porcine VSMCs (Fig. 2E), Aβ dose dependently inhibited NO (10 µM DEA/NO) stimulated adhesion of VSMC to collagen coated wells (Fig. 2F). 10 µM Aβ inhibited NO-stimulated VSMC adhesion by 40±12%.

Thrombin-induced platelet aggregation is potently delayed by NO-cGMP signaling [43]. Under standard high sheer conditions, thrombin induced aggregation of washed human platelets was delayed by the addition of 10 nM DEA/NO (Fig. 2G). This delay was reversed by pre-treating the platelets with 10 µM Aβ prior to the addition of 10 nM DEA/NO. The same effect of Aβ was also observed when platelet aggregation was assessed under low sheer conditions (data not shown). Thus, consistent with the inhibition of NO and sGC activator induced stimulation of cGMP production, Aβ inhibits at least two functional outcomes of cGMP signaling in cells.

### Aβ inhibition of NO signaling requires CD36

**Figure 3:**
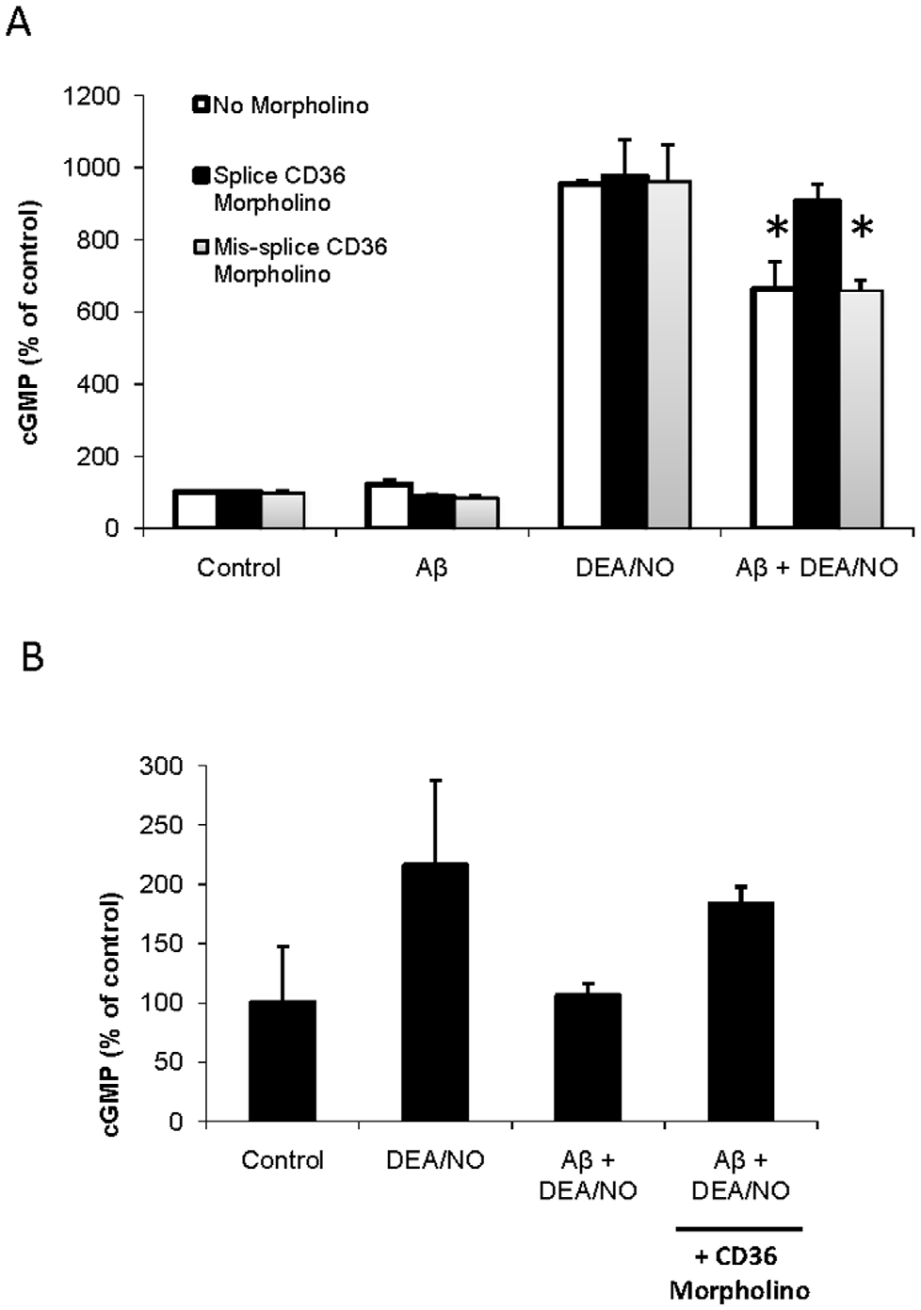
Aβ inhibition of NO signaling is dependent on CD36. **A** Jurkat cells or **B** BAEC were incubated with 10 µM CD36 antisense morpholino or 10 µM 5-mis-splice CD36 control morpholino for 48 hrs. Following CD36 knockdown, cells were pretreated with 10 µM Aβ followed by 10 µM DEA/NO. Following treatment, cell were lysed and assayed for cGMP production. n = 3, * denotes P<0.05.

CD36 has been implicated as a receptor/mediator for other targets of Aβ signaling [26], [28]. Data in Figures 1A–C suggest that Aβ directly binds to CD36 based on its inhibiting cellular uptake of the CD36 ligand myristic acid, but we cannot exclude that Aβ inhibits myristate update indirectly by binding to another CD36-associated protein. Previous results from our lab have shown that binding of recombinant type 1 repeats of TSP1, a synthetic peptide derived from this domain of TSP1, or a related peptidomimetic to CD36 is sufficient to inhibit both myristate uptake and NO-cGMP signaling [25], [32]. To examine whether Aβ inhibition of cGMP signaling requires CD36, BAEC and Jurkat cells were pretreated with a splice-blocking CD36 morpholino oligonucleotide. In both cell types this knockdown resulted in decreased Aβ inhibition of NO induced cGMP accumulation (Fig. 3A, B). Nearly complete reversal of Aβ inhibition occurred when CD36 expression was suppressed in HUVEC. Therefore, unlike the inhibitory activity of TSP1, CD36 expression is necessary for Aβ to inhibit sGC activation in these cells.

### Aβ inhibition of NO signaling also requires CD47

**Figure 4:**
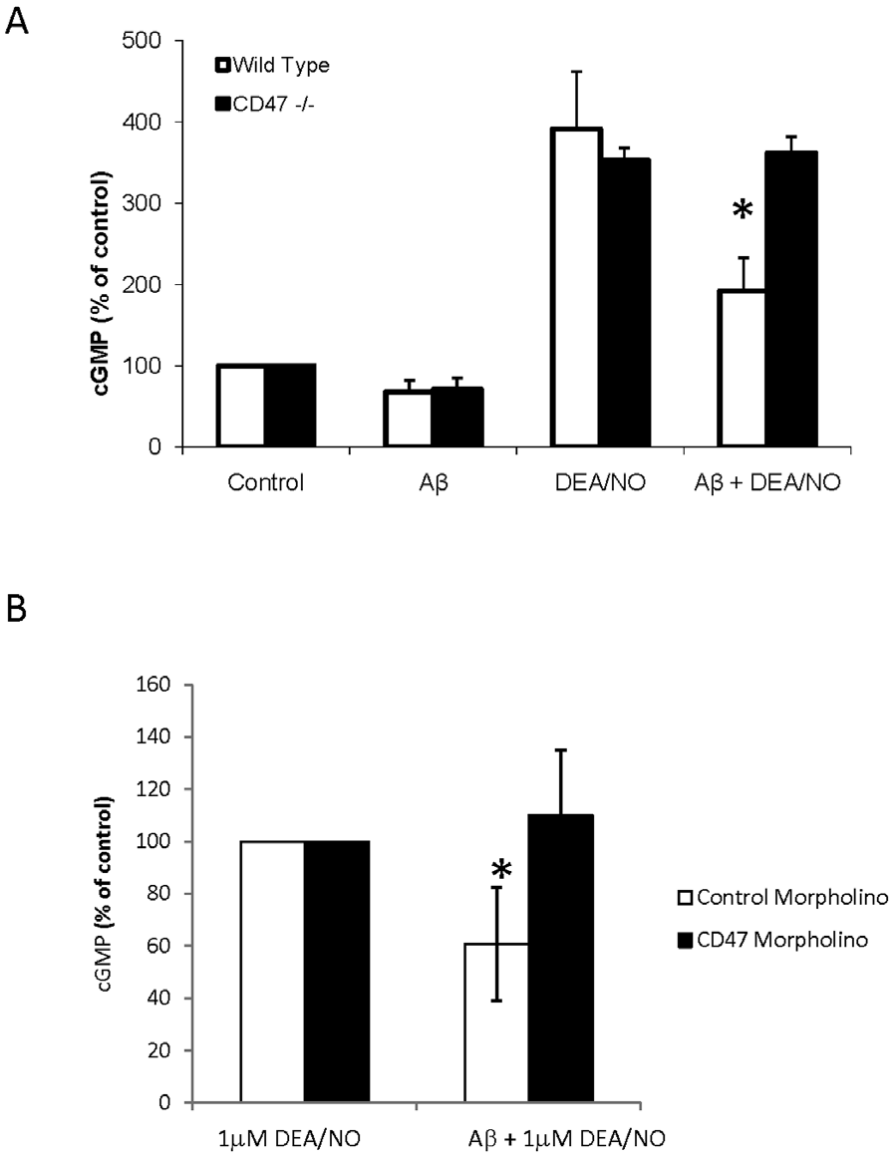
Aβ inhibition of NO signaling is dependent on CD47. **A** Wild type or CD47−/− primary murine lung endothelial cells were pretreated with 10 µM Aβ followed by 10 µM DEA/NO. **B** Wild type Jurkat cells were incubated with 10 µM CD47 antisense morpholino or 10 µM of a 5 base mismatched CD47 control morpholino for 48 hrs. Following CD47 knockdown, cells were pretreated with 10 µM Aβ followed by 1 µM DEA/NO. Following treatment, cell were lysed and assayed for cGMP production. n = 3, * denotes P<0.05.

Although ligation of CD36 is sufficient to inhibit cGMP signaling, this inhibition is lost in cells lacking CD47 [25]. Therefore, CD47 is necessary for CD36-mediated inhibition of cGMP signaling. In contrast, TSP1 inhibition of cGMP signaling via of its high affinity binding to CD47 does not require CD36 [39]. This suggests that modulation of cGMP signaling by CD36 ligands is mediated by cross-talk with CD47. Consistent with the BAEC data described above, wild-type murine vascular cells expressing CD47 and treated with 10 µM DEA/NO showed a 4-fold increase in intracellular cGMP production that was inhibited by the addition of Aβ (Fig. 4A). In contrast, Aβ failed to suppress the increase in the cGMP production induced by DEA/NO in murine CD47−/− lung endothelial cells. Conversely, suppressing CD47 expression in Jurkat T-cells using a CD47-specific morpholino abolished the effect of Aβ on NO-stimulated cGMP accumulation (Fig. 4B). Thus, two independent approaches confirm that CD47 is necessary for Aβ to suppress NO signaling.

### Aβ inhibition of NO signaling is TSP1 independent

**Figure 5:**
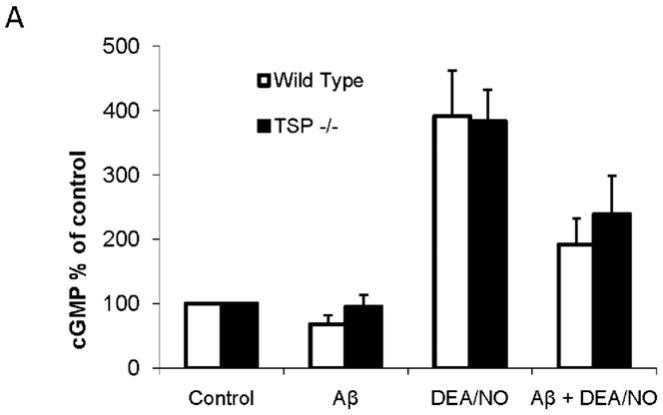
Aβ inhibition of NO signaling is not dependent on TSP1. **A** Wild type or TSP1−/− primary murine lung endothelial cells were pretreated with 10 µM Aβ followed by 10 µM DEA/NO. Following treatment, cell were lysed and assayed for cGMP production. n = 3, * denotes P<0.05.

TSP1 is known to inhibit NO signaling by suppressing sGC activity through CD47, in the same manner through which we observed Aβ to inhibit NO signaling. To rule out the possibility that the observed Aβ inhibitory activity is due to or influenced by mobilization of endogenous TSP1, we used TSP1 null cells to evaluate Aβ inhibition of NO signaling. As shown in Figure 5, in wild type primary murine lung endothelial cells bearing TSP1, the addition of DEA/NO increased intracellular cGMP production, while treatment with Aβ inhibited DEA/NO stimulated cGMP production, as expected. In the corresponding TSP1 null cells, a similar pattern was observed in which Aβ inhibited an increase in cGMP levels caused by DEA/NO and thus inhibited sGC activity. Therefore, Aβ inhibition of NO signaling is not an artifact of modulating endogenous TSP1.

## Discussion

Although previous studies have suggested that Aβ is involved in the inhibition of NO-induced processes such as hippocampal long term potentiation [21], vascular tone [23], and CREB phosphorylation (18), the molecular mechanism through which inhibition of NO signaling occurs was not defined. Identifying how Aβ is able to suppress the NO signaling pathway is important in the development of therapeutics aimed at slowing Alzheimer's disease pathogenesis because enhancing neuronal NO has the potential to protect against neurodegeneration. Here, we provide evidence that Aβ inhibits the NO signaling pathways through its interactions with CD36, which causes a CD47-dependent decrease in sGC activity and cGMP production. This inhibition was reproduced in VSMC, endothelial cells, and T cells and prevents both NO- and drug-mediated activation of sGC. We also showed that Aβ can inhibit uptake of free fatty acids via CD36, which was previously established to regulate NO synthesis in vascular cells [37], [44].

**Figure 6:**
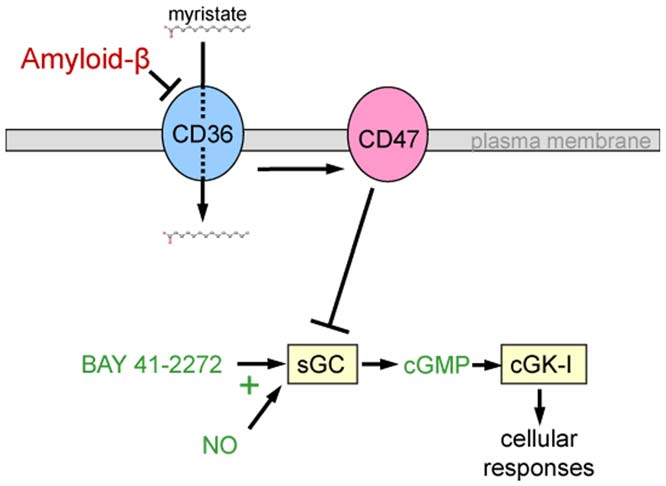
Proposed model of Aβ inhibition of cGMP production. Aβ binds directly to CD36 to inhibit uptake of free fatty acids. In the presence of CD47, CD36 engagement transduces an inhibitory signal to sGC, limiting its activation and production of cGMP.

The activity of Aβ to inhibit NO/cGMP signaling in vascular and T cells suggests that pathological accumulation of Aβ can play a key role in limiting the NO signaling pathway (7). Previously, various downstream targets of NO have been shown to be inhibited by Aβ (18, 19). Here, we provide a link between Aβ and NO signaling by showing that all the downstream inhibitory responses could result from suppression of sGC activity by Aβ (Figure 6). We further report that Aβ offsets increases in cGMP levels caused by both NO donors and synthetic sGC activators, indicating that Aβ can inhibit sGC independent of its NO-binding heme prosthetic group. This is important because others have shown that sGC can be inhibited by oxidizing the Fe^2+^ in this heme.

Our results further show that decreased sGC activity and NO signaling caused by Aβ are dependent on the presence of CD47. Previously, Aβ fibrils were proposed to attach to microglial cells by interacting with a cell surface receptor complex that includes CD47, α_6_β_1_ integrin, and CD36 [3]. While the evidence for functional involvement of CD36 in Aβ signaling was strong, the evidence for CD47 binding was only inferred based on sensitivity to 4N1K, a thrombospondin-1-based peptide with both CD47-dependent and CD47-independent activities [45], [46]. The ability of Aβ to directly interact with integrins is also controversial. An Arg-His-Asp sequence in Aβ was shown to be recognized by α_5_β_1_ integrin and mediate its uptake and degradation [47]. However, another study concluded that integrin antagonists increase uptake of Aβ and increase its neurotoxicity in brain tissue [48]. Beta-2 integrins have also been identified as Aβ receptors [49], and α_2_β_1_ and α_v_β_1_ were implicated in fibrillar amyloid deposition [50]. Therefore, it is unlikely that α_6_β_1_ is a specific integrin receptor for Aβ that could account for its inhibition of NO signaling.

We propose a revised version of the Aβ binding model presented by Bamberger *et al*. Aβ inhibition of cGMP-dependent signaling requires CD47, but Aβ binding may depend more on the interaction with the scavenger receptor CD36, which is well known for its promiscuous binding to a variety of extracellular ligands (reviewed in [51]). Binding of Aβ to CD36 or to an associated molecule generates an inhibitory signal that is probably transduced via CD47 (Figure 6). This model is consistent with the requirement for both CD36 and CD47 and the inability of Aβ to displace SIRPα, a specific CD47 ligand. CD47 also plays a role in Aβ inhibition of kinase-based signal transduction cascades [26] and Vav1 activation [28], but like inhibition of sGC activation, these responses may be independent of direct binding between Aβ and CD47.

Aβ regulation of NO signaling through CD47 differs slightly from TSP1 inhibition of NO signaling in vascular cells [25]. CD47 is a high affinity receptor for TSP1, and this binding inhibits NO signaling by suppression of sGC activation and a decrease in cGMP levels (20, 22). In contrast, Aβ does not interact directly with CD47. TSP1 can also bind to CD36, but the affinity of this interaction is lower. Thus, CD47 is the dominant signaling receptor for physiological concentrations of TSP1. Aβ appears to not bind to CD47, but CD47 is necessary for Aβ's inhibition of sGC activation. The details of how CD47 contributes to CD36-mediated Aβ signaling to decrease sGC activation remain to be defined.

Recognizing that Aβ inhibits NO signaling by suppressing sGC activity through CD36 and CD47 may clarify Aβ's role in Alzheimer's disease pathogenesis and could lead to novel therapeutics aimed at limiting the adverse effects of Aβ in the brain. As mentioned previously, NO can protect against neuron degeneration and inflammation, but its signaling is inhibited by Aβ at the level of sGC activation. Thus, elevating the steady state NO levels would be more effective in combination with an agent targeting CD47 to relieve the block at sGC. Realizing that this inhibition occurs through CD47 and CD36, therapeutic approaches could be developed to block Aβ signaling through these receptors to rescue the NO signaling pathway.

In addition to providing a mechanistic basis for the recognized deficiencies in NO/cGMP signaling associated with Alzheimer's disease pathogenesis, our data may have implications for peripheral vascular disease in light of the documented Aβ accumulation in diseased peripheral as well as cerebral blood vessels. Consistent with the inhibitory activities of Aβ we describe using large vessel endothelial and VSMC, Aβ was previously shown to inhibit vascular reactivity in isolated rabbit and rat aorta [33], [52]. Furthermore, the secretion of Aβ by activated platelets during aging and hypercholesterolemia may expose the peripheral vasculature to this NO antagonist and account for part of the NO insufficiency and insensitivity of these diseases [53], [54].

Mutations resulting in CD36 deficiency occur in humans and some strains of spontaneously hypertensive rats. Humans with CD36 deficiency exhibit hyperlipidemia, increased remnant lipoproteins, impaired glucose metabolism based upon insulin resistance, and mild hypertension [55]. Based on our observation that Aβ impairs the fatty acid translocase activity of CD36, the accumulation of vascular-associated Aβ in Alzheimer's disease could cause a local functional CD36 deficiency by blocking lipid and, potentially, oxidized lipoprotein uptake via this receptor. In addition to perturbing vascular NO signaling via CD36, this direct effect of Aβ on CD36 translocase function merits further study.

## Materials and Methods

### Cells and reagents

Bovine aortic endothelial cells (BAECs) prepared from fresh aortic segments[56] and human umbilical vein endothelial cells (HUVEC) obtained from Lonza (Walkersville, MD) were cultured in endothelial growth medium (EGM; Lonza, Walkersville, MD) in 5% CO_2_ at 37°C, and were used at passages 3–8. Jurkat human T-lymphoma cells[57] were cultured in RPMI 1640 (Invitrogen) with 10% FBS, glutamine, and penicillin/streptomycin. Cells were kept at a density of between 1×10^5^ and 5×10^5^ and used between passages 7 and 20. Primary mouse endothelial cells were obtained from the lungs of wild-type, TSP1-null, and CD47-null C57BL/6 mice after sacrifice[39], and grown in EGM as above. BV2 immortalized murine microglial cells[58] (a gift of Dr. Sandra Rempel, Hermelin Brain Tumor Center, Henry Ford Health System, Detroit, MI) were maintained in DMEM (Invitrogen) with 10% FBS, glutamine, and penicillin/streptomycin. Porcine vascular smooth muscle cells (white hairless Yucatan miniature pig, as described in [59]) and human aortic vascular smooth muscle cells (VSMC, Lonza) were cultured in SMGM2 (Lonza; Walkersville, MD) and were used at passages 3–8. DEA/NO was provided by Dr. Larry Keefer (NCI, Frederick, MD). BAY 41–2272 and IBMX (3-isobutyl-1-methylxanthine) were obtained from Calbiochem (La Jolla, CA). Aβ(1–42) peptides were purchased from Anaspec (San Jose, CA). Fibrillar Aβ was made by incubating peptides in sterile distilled water for 1 week at 37°C. Extracellular domain of SIRPα fused to a modified human Fc domain was prepared and labeled with ^125^I as previously described [39], [60]. Anti-CD47 antibody B6H12 was purchased from Abcam (Cambridge, UK). Care and handling of animals was in accordance with and approved by Animal Care and Use Committees of the National Cancer Institute (Protocol LP-012).

### Intracellular cGMP assay

BAEC, porcine VSMC, or mouse primary cells were plated in 6-well plates. The cells were grown in serum containing media until reaching 80% confluence, at which time they were serum starved overnight in endothelial basal medium (EBM; Lonza) or smooth muscle basal medium (SMBM; Lonza) with 0.1% bovine serum albumin (BSA; Sigma-Aldrich, St Louis, MO). Jurkat cells were assayed at 5×10^5^ cells per condition in 0.5 ml basal media (RPMI, glutamine, penicillin/streptomycin, 0.01% BSA). The relevant cells were pre-incubated for 15 min with the indicated concentrations of Aβ and then treated with DEA/NO for 2 min at room temperature. The cells were then lysed, and total intracellular cGMP levels were measured via immunoassay using a cGMP kit (GE/Amersham Healthcare, Amersham, United Kingdom) according to the manufacturer's instructions. A μBCA protein assay (Thermo Scientific, Rockford, IL) was performed to determine the protein concentration for each of the samples. cGMP levels were normalized based on the amount of protein present.

Experiments using BAY 41–2272 followed the same above procedure, except 10 µM of BAY 41–2272 in DMSO was used instead of DEA/NO. In the experiments involving BAY 41–2272, dimethyl sulfoxide (DMSO; Sigma) was also added to control wells.

### [^3^H]-myristate uptake assay

The [^3^H]myristic acid uptake assays were performed using 80–90% confluent HUVEC, human microglial, and human aortic VSMC cells (5×10^5^ cells/well) in 24-well culture plates (Nunc, Denmark). Trace amounts of [^3^H]myristic acid (5 µCi/ml, 0.9 µM) mixed with 9.1 µM nonradioactive myristic acid were dissolved in a FAF BSA solution at a myristic acid/BSA molar ratio of 1∶2. Cells were incubated in medium with treatment agents for the indicated time intervals at 37°C. The uptake was stopped by removal of the solution followed by the addition of chilled 0.9% NaCl with 0.5% BSA. The stop solution was discharged, and the cells were washed again with stop solution. Cells were lysed by adding 0.2 M NaOH (200 µl/well) and incubated for 2 h at 37°C. On completion of solubilization, 0.2 M HCl in 1.5 m Tris-HCl (200 µl) was added to each well. Radioactivity was determined in 10 ml of Ecoscint A (National Diagnostics, Atlanta, GA) using a 1900CA liquid scintillation counter (Packard Instrument Co.).

### ^125^I-SIRPα-Fc binding assay

1×10^6^ Jurkat T-cells in PBS with cations and 0.1% BSA were incubated with 0.4 ug/ml ^125^I-SIRPα and the indicated concentrations of Aβ peptide or CD47-specific monoclonal antibody (B6H12) shaking at 25°C for 1hour. Cells were separated from unbound ^125^I-SIRPα by centrifugation through silicone oil (nyosil M25, Nye Co, New Bedford, MA). Cell-bound radioactivity was quantified using a PerkinElmer Life Sciences gamma counter. Data are represented as percent of total counts (no cells), n = 3.

### VSMC adhesion assay

Cell adhesion was carried out in 96-well plates (Nunc, Denmark). After precoating wells with type I collagen (3 µg/ml), porcine VSMC were plated at a density of 1×10^4^ cells/well in SMBM containing 0.1% BSA and treatment agents and incubated in 5% CO_2_ for 1 h. Wells were washed with PBS, and the cells were fixed with 1% glutaraldehyde for 10 min, washed, and stained with 1% crystal violet for 20 min. Excess stain was rinsed away, the cells were extracted with 10% acetic acid, and the plates were read at 570 nm.

### Preparation of human platelets

Platelets were obtained obtained as byproducts from healthy volunteers through the NIH department of transfusion medicine blood bank. “Byproducts” of transfusion donations are provided anonymously, without any identifying code or number. The link between the donor and the product is irreversibly destroyed by the DTM. As such, distribution of these products is exempt from the need for IRB approval. Platelets were pelleted from platelet-rich plasma by centrifugation for 10 min at 200 g. They were then resuspended in acid citrate dextrose (ACD; 85 mM citric acid, 65 mM sodium citrate, 100 mM glucose, pH 5.1) at a ratio of 1∶10 at room temperature. Platelets were pelleted again and resuspended in 10 ml of Tyrode buffer (137 mM NaCl, 3 mM KCl, 12 mM NaHCO_3_, 0.3 mM NaHPO_4_, 2 mM CaCl_2_, 1 mM MgCl_2_, 5.5 mM glucose, 5 mM N-2-hydroxyethylpiperazine- N-2-ethanesulphonic acid (HEPES), 3.5 mg/ml BSA, pH 7.4). The final platelet number was adjusted to 6.5×10^5^ platelets/ml in a cuvette containing 500 µl of Tyrode buffer.

### Platelet aggregation assay

Aggregation of human platelets under high shear conditions was assessed using a standard optical aggregometer (Lumi- Dual Aggregometer; Chrono-Log, Havertown, PA, USA) at 37°C and 1200 rpm in a volume of 500 mL buffer with a final platelet concentration of 6.5×10^5^ platelets/ml over a 5 min interval. Preincubation with Aβ (10 µM) was for 5 min prior to addition of DEA/NO (0.01 µM) or vehicle control, which were incubated 5 min prior to the initiation of aggregation with thrombin (0.2 U).

### Statistical analysis

All assays were repeated at least in triplicate and some are normalized to percents of control in order to account for the differences in cell count and conditions between trials. The results were expressed as means ± SD or shown as representative data. Statistical significance was determined by the Student t test. A *P-*value less than 0.05 was regarded as statistically significant.
